# Cysts and tumors of the jaws treated by marsupialization: A description of 4 clinical cases

**DOI:** 10.4317/jced.55563

**Published:** 2019-06-01

**Authors:** Sondes Briki, Wael Elleuch, Fathi Karray, Mohamed Abdelmoula, Issam Tanoubi

**Affiliations:** 1 MD. Maxillofacial Surgery Department, Faculty of medicine of Sfax, Tunisia; 2 MD, MA. (ed). Centre d’Apprentissage des Attitudes et Habiletés Cliniques (CAAHC), Simulation center of the Université De Montréal

## Abstract

Since the 4th edition of the World Health Organization’s Classification of Head and Neck Tumours was published in January of 2017, the keratocystic odontogenic tumor is back into the cyst category as odontogenic keratocyst (OKC). Depending on the size of the cyst, its location and the patients’ age, several treatment options are available: curettage, enucleation, radical treatment and marsupialization. The marsupialization is a conservative technique used in early tumor stages, as curative treatment for the odontogenic cyst. Despite its disadvantages and controversies, the marsupialization remains an interesting therapeutic choice in the case of large cysts, or in very young or old patients. We describe, in this article, four clinical cases of odontogenic cysts. We report the surgical management and the subsequent evolution of the patients. The discussion focuses on the indications, advantages and limitations of the odontogenic cyst’s marsupialization. We review the specific conditions of the odontogenic cysts that could make the marsupialization the optimal therapeutic option. In our cases, the marsupialization proved to be a conservative technique which allowed the respect of neighboring anatomical structures, particularly in the case of large cysts, but requires prolonged clinical and radiological monitoring. Pathological entity for our cases was different. Thus, the treatment outcome may be different too. This series is very small and the reader should be cautious about drawing broad conclusions regarding the optimal therapeutic choice.

** Key words:**Marsupialization, odontogenic cyst.

## Introduction

Since the 4th edition of the World Health Organization’s Classification of Head and Neck Tumours was published in January of 2017 (1), the keratocystic odontogenic tumor is back into the cyst category as odontogenic keratocyst (OKC). Depending on the size of the cyst, its location and the patients’ age, several treatment options are available: curettage, enucleation, radical treatment and marsupialization. The marsupialization is a surgical technique used as the first stage or the definitive treatment for the odontogenic keratocyst (OKC). It was first described by Partsch in the late 19th century for the cystic lesions of the jaws (2,3) which treatment represents a challenge for surgeons especially in the large tumors. This technique is based on the externalization of the cyst through the creation of a surgical window in the buccal mucosa and in the cystic wall. Their borders are then sutured to create an opened cavity that communicates with the oral cavity (3). We describe four cases of a successfully used marsupialization and we highlight the advantages of this conservative technique. We also review its limitations.

## Case Report

All the patients, or the parents (case 1) consented to the publication of these cases without disclosure of their identity. The  Table 1 describes the major clinical characteristic, radiological features and histological type of each cyst and tumor.

**Table 1:**
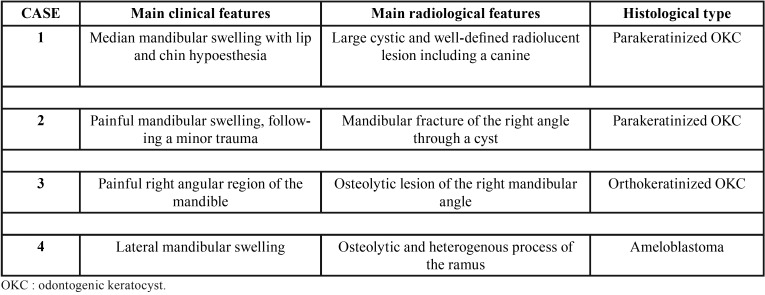
Summary of the different presentations of the described mandibular cysts and tumor.

**Figure 1:**
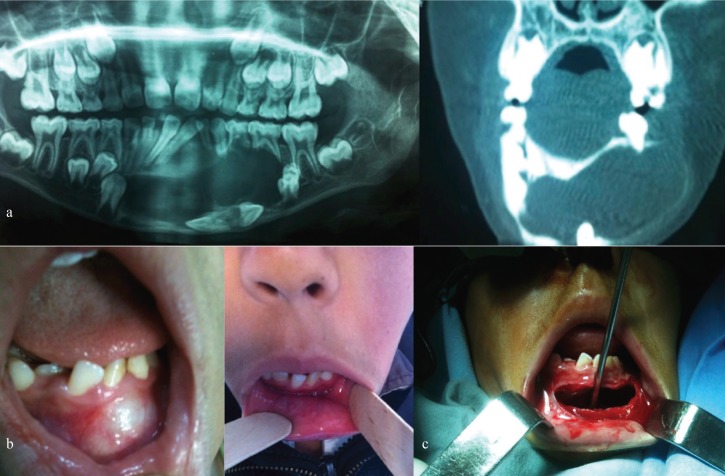
a- Radiologic data: Orthopantogram (OPG) and computed tomography scan with giant cyst of the mandible involving an included canine. b: Examination before marsupilazation (a), and after 6 months (b). c: Intraoperative view after opening of cystic cavity.

Case 1:

A 10-years-old boy was referred by his dentist for median mandibular swelling with lip and chin hypoesthesia in the last 4 months. The intra oral examination showed a soft tumor filling the vestibule with no dental mobility. The panoramic radiograph (OPG) and the CT scan showed a large cystic and well-defined radiolucent lesion including a canine. The OKC was histologically parakeratinized. The treatment consisted in the marsupialization with weekly changed gauze in the cavity, the extraction of included tooth and the biopsy of cystic wall which confirmed the diagnosis of keratocyst. Parents have accepted this long follow-up period, despite the long-distance constraints of each follow-up appointment, in order to avoid the aggressive treatment for the growing child. The follow-up after 6 months showed no sign of recurrence with good radiologic ossification (Fig. 1).

**Figure 2:**
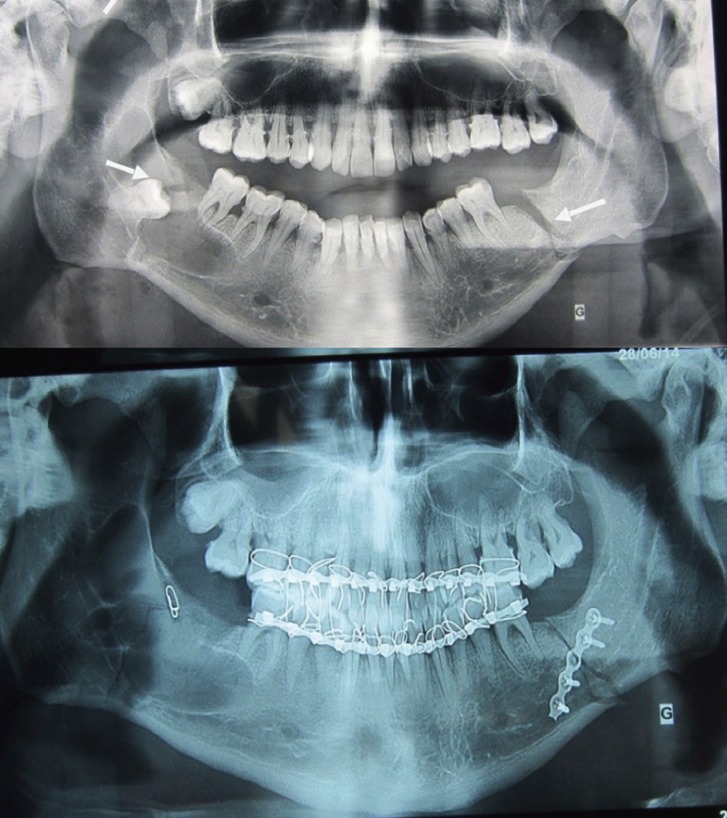
Orthopantogram and CT scan showing bifocal mandibular fracture and mandibular cyst. Orthopantogram shows an evident fracture of the left mandibular rim and cystic cavity in the right side. Orthopantogram after the surgery.

Case 2:

A 40 years old man complains about a major painful mandibular swelling, following a minor trauma of his mandibula. The OPG and CT scan showed a mandibular fracture of the right angle through a cyst including a molar and extending to the ramus, and another fracture of the left mandibular branch. This cyst was a parakeratinized OKC. The treatment consisted in the reduction and fixation of the right mandibular fracture with the cyst marsupialization, with gauze in the cavity, changed weekly. The biopsy of cystic wall confirmed the diagnosis of periapical cyst. The left fracture was treated by the osteosynthesis and maxillomandibular fixation for one month. In a second stage, the curettage of the cavity and the enucleation of the cyst was made after 3 months. We chose this treatment to avoid osteosynthesis of a fragile cystic cavity, with a high risk of infection. The evolution after 6 months was favorable, with a good ossification of the mandibular bone (Fig. 2).

Case 3:

A 68 years old women complains about painful right angular region of the mandible in the last 7 months. The OPG and CT scan showed an osteolytic lesion of the right mandibular angle, diagnosed histologically as orthokeratinized OKC. The treatment consisted in the marsupialization with biopsy for histological examination, diagnosing a keratocyst. This short-term operative procedure was performed on an ambulatory basis, thus avoiding an invasive surgery in an elderly subject as well as the associated post-operative pain and cognitive disorders.

**Figure 3:**
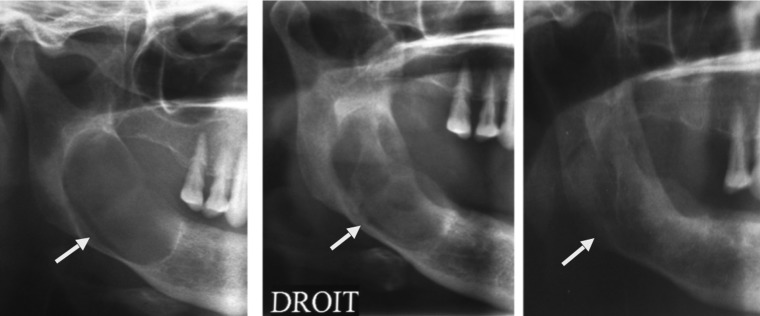
Radiologic evolution on orthopantogram. Initial cystic lesion of the right mandible. Reduction of the cystic size 3 months after marsupialization.Complete ossification of the right mandibular 6 months after marsupialization.

The follow-up showed a good ossification after 3 and 6 months with no sign of recurrence (Fig. 3).

Case 4.

 A 32 years old man presented a lateral mandibular swelling in the past 10 months. The CT scan showed an osteolytic and heterogenous process of the ramus. The treatment consists of the marsupialization with biopsy (concluding on an ameloblastoma) and secondary enucleation after the decreasing the size of the lesion (after 8 months). This two-stage treatment has been judged as best patient option to avoid radical treatment with mandibulectomy in our young patient. The 4 years follow-up showed good ossification after enucleation with no sign of recurrence.

## Discussion

Although the marsupialization and the decompression of cysts are two terms interchangeably used in many articles, they have a different technical meaning. The decompression includes all the options used to reduce the pressure from within a cyst, however, the marsupialization lies in the conversion of the cyst into an open pouch, that communicates with oral cavity (2,3). Many devices can be used to maintain this opened in mouth window such as tubes, gauzes, obturators and acrylic stent (4,5).

The marsupialization is also described as a Partsch I procedure. The Partsch II procedure is enucleation and primary closure (4).

The marsupialization has the purpose to relieve the pressure into the cyst which results of the decrease of the expression of IL-1α and other inflammatory cytokines, allowing new bone to fill the defect (3,4,6).

Ninomiya *et al.* (6) noted also, by immunohistochemistry in the lining epithelium of odontogenic keratocysts after the marsupialization, that the thickness of epithelium was increased, and the keratinized epithelium was changed to a hyperplasic and non-keratinized stratified squamous epithelium.

In recent years, many articles have reported the marsupialization as one of the suitable treatment options for the cystic lesions of the jaws (7,8). The marsupialization was described as a conservative treatment modality for specific cystic lesions such as dentigerous cysts (9,10), radicular cysts (11,12), keratocystic odontogenic tumour (13,14) and cystic ameloblastoma (15,16).

In the presence of a giant mono cystic lesion or in case of extreme age patients, as suggested in our first and third patient, the marsupialization specifically allows conservative treatment for the aggressive cystic lesions and avoids more aggressive radical surgery.

The technique of marsupialization has always been called “less aggressive” for several reasons. It minimizes the cyst (13,15-17), promotes the eruption of the involved teeth and maintains the developing dentition by minimizing any disturbance, for example, fibrous scars formation, to future dental development (9,18-20), well-illustrated by our first described case. For these reasons, marsupialization should be among the first therapeutic options in the pediatric population, with extensive cyst. Also, when compared with the often mutilating radical primary cystectomies or resection methods, it minimizes the damage of the important anatomical tissue nearby, including inferior alveolar nerve and sinus, minimizes the damage of the bone tissue, stimulates osteogenesis (15), and reduces the risk of to pathologic fracture of mandible (13,14,21).

The marsupialization reduces the recurrence of the lesions and ultimately eliminates medication and hospitalization costs for most cases (2).

The use of marsupialization is limited by its long healing period and the discomfort of the patient, especially at the early stages of the marsupialization (13,14). Indeed, the irrigation of the cyst cavity is obligatory and containing twice a day and the device used to maintain the opening of the cyst needs to be changed every week. Needless to say that it requires highly cooperative patients, and parents, which have a major impact on the success rate of this treatment plan.

After the disappearance of the cyst cavity, the management plan may need a second surgical, non-invasive, intervention to eliminate the residual pathological tissue (13,17).

In pediatric patients, a longer follow-up period is required for to ensure the adequate eruption of the teeth and the absence of recurrence (8,22), especially with Keratocyst odontogenic cyst, as its recurrence rate is not affected by the marsupialization (13,17). Our patients have accepted the constraints of this long-term therapeutic method to avoid radical and heavy surgery in certain situations.

The main purpose of our case description is to focus on marsupialization of cystic lesions. But the variability of these lesions and the multiple histological types (23) like inflammatory cysts, developmental cysts, benign tumors (keratocystic odontogenic tumor, ameloblastoma, adenomatoid odontogenic tumor) or malignant tumors (clear cell odontogenic carcinoma, ameloblastic carcinoma and fibrosarcoma), do not allow to generalize this surgical management to all the mandibular cystic lesions.

## Conclusions

Marsupialization could be used as single surgical procedure or combined with other treatment modalities for cystic lesions in different sites of the jaws. It has been accepted as conservative and non-invasive surgical option especially in extreme age patients and in the presence of giant cyst. The containing treatment plan could be one of the major factors limiting the use of the marsupialization. The long-term observation is required to ensure its effectiveness.

## References

[1] Wright JM, Vered M (2017). Update from the 4th Edition of the World Health Organization Classification of Head and Neck Tumours: Odontogenic and Maxillofacial Bone Tumors. Head Neck Pathol.

[2] Borgonovo AE, Di Lascia S, Grossi G, Maiorana C (2011). Two-stage treatment protocol of keratocystic odontogenic tumour in young patients with Gorlin-Goltz syndrome: marsupialization and later enucleation with peripheral ostectomy. A 5-year-follow-up experience. Int J Pediatr Otorhinolaryngol.

[3] Rui H, Hongzhi Z (2013). Articles of marsupialization and decompression on cystic lesions of the jaws: A literature review. Journal of Oral and Maxillofacial Surgery, Medicine, and Pathology.

[4] Pogrel MA (2005). Treatment of keratocysts: the case for decompression and marsupialization. J Oral Maxillofac Surg.

[5] Carter LM, Carr P, Wales CJ, Whitfield PH (2007). Customised stents for marsupialisation of jaw cysts. Br J Oral Maxillofac Surg.

[6] Ninomiya T, Kubota Y, Koji T, Shirasuna K (2002). Marsupialization inhibits interleukin-1alpha expression and epithelial cell proliferation in odontogenic keratocysts. J Oral Pathol Med.

[7] Lee ML, Prepageran N, Subha ST (2004). Dentigerous cyst of the maxillary sinus in a child. Med J Malaysia.

[8] Pogrel MA, Jordan RC (2004). Marsupialization as a definitive treatment for the odontogenic keratocyst. J Oral Maxillofac Surg.

[9] Jones TA, Perry RJ, Wake MJ (2003). Marsupialization of a large unilateral mandibular dentigerous cyst in a 6-year-old boy--a case report. Dent Update.

[10] Gondim JO, Neto JJ, Nogueira RL, Giro EM (2008). Conservative management of a dentigerous cyst secondary to primary tooth trauma. Dent Traumatol.

[11] Loushine RJ, Weller RN, Bellizzi R, Kulild JC (1991). A 2-day decompression: a case report of a maxillary first molar. J Endod.

[12] Balaji Tandri S (2010). Management of infected radicular cyst by surgical decompression. J Conserv Dent.

[13] Maurette PE, Jorge J, de Moraes M (2006). Conservative treatment protocol of odontogenic keratocyst: a preliminary study. J Oral Maxillofac Surg.

[14] Tolstunov L, Treasure T (2008). Surgical treatment algorithm for odontogenic keratocyst: combined treatment of odontogenic keratocyst and mandibular defect with marsupialization, enucleation, iliac crest bone graft, and dental implants. J Oral Maxillofac Surg.

[15] Nakamura N, Higuchi Y, Mitsuyasu T, Sandra F, Ohishi M (2002). Comparison of long-term results between different approaches to ameloblastoma. Oral Surg Oral Med Oral Pathol Oral Radiol Endod.

[16] Huang IY, Lai ST, Chen CH, Chen CM, Wu CW, Shen YH (2007). Surgical management of ameloblastoma in children. Oral Surg Oral Med Oral Pathol Oral Radiol Endod.

[17] Nakamura N, Mitsuyasu T, Mitsuyasu Y, Taketomi T, Higuchi Y, Ohishi M (2002). Marsupialization for odontogenic keratocysts: long-term follow-up analysis of the effects and changes in growth characteristics. Oral Surg Oral Med Oral Pathol Oral Radiol Endod.

[18] Murakami A, Kawabata K, Suzuki A, Murakami S, Ooshima T (1995). Eruption of an impacted second premolar after marsupialization of a large dentigerous cyst: case report. Pediatr Dent.

[19] Delbem AC, Cunha RF, Afonso RL, Bianco KG, Idem AP (2006). Dentigerous cysts in primary dentition: report of 2 cases. Pediatr Dent.

[20] Koca H, Esin A, Aycan K (2009). Outcome of dentigerous cysts treated with marsupialization. J Clin Pediatr Dent.

[21] Zhao YF, Wei JX, Wang SP (2002). Treatment of odontogenic keratocysts: a follow-up of 255 Chinese patients. Oral Surg Oral Med Oral Pathol Oral Radiol Endod.

[22] Bodner L (2002). Cystic lesions of the jaws in children. Int J Pediatr Otorhinolaryngol.

[23] Bilodeau EA, Collins BM (2017). Odontogenic Cysts and Neoplasms. Surg Pathol Clin.

